# 3D IMAGING OF THE MITOCHONDRIAL REDOX STATE OF RAT HEARTS UNDER NORMAL AND FASTING CONDITIONS

**DOI:** 10.1142/S1793545813500454

**Published:** 2013-10-16

**Authors:** HE N. XU, RONG ZHOU, LILY MOON, MIN FENG, LIN Z. LI

**Affiliations:** * Department of Radiology, University of Pennsylvania, Philadelphia, PA 19104, USA; † Britton Chance Laboratory of Redox Imaging, Johnson Research Foundation, Department of Biochemistry and Biophysics, University of Pennsylvania, Philadelphia, PA 19104, USA; ‡ Institute of Translational Medicine and Therapeutics, Perelman School of Medicine, University of Pennsylvania, Philadelphia, PA 19104, USA

**Keywords:** Mitochondrion, metabolism, bioenergetics, cardiomyocyte, NADH, flavoproteins, Fp, FAD, caloric restriction, food deprivation

## Abstract

The heart requires continuous ATP availability that is generated in the mitochondria. Although studies using the cell culture and perfused organ models have been carried out to investigate the biochemistry in the mitochondria in response to a change in substrate supply, mitochondrial bioenergetics of heart under normal feed or fasting conditions has not been studied at the tissue level with a sub-millimeter spatial resolution either *in vivo* or *ex vivo*. Oxidation of many food-derived metabolites to generate ATP in the mitochondria is realized through the NADH/NAD^+^ couple acting as a central electron carrier. We employed the Chance redox scanner — the low-temperature fluorescence scanner to image the three-dimensional (3D) spatial distribution of the mitochondrial redox states in heart tissues of rats under normal feeding or an overnight starvation for 14.5 h. Multiple consecutive sections of each heart were imaged to map three redox indices, i.e., NADH, oxidized flavoproteins (Fp, including flavin adenine dinucleotide (FAD)) and the redox ratio NADH/Fp. The imaging results revealed the micro-heterogeneity and the spatial distribution of these redox indices. The quantitative analysis showed that in the fasted hearts the standard deviation of both NADH and Fp, i.e., SD_NADH and SD_Fp, significantly decreased with a *p* value of 0.032 and 0.045, respectively, indicating that the hearts become relatively more homogeneous after fasting. The fasted hearts contained 28.6% less NADH (*p* = 0.038). No significant change in Fp was found (*p* = 0.4). The NADH/Fp ratio decreased with a marginal *p* value (0.076). The decreased NADH in the fasted hearts is consistent with the cardiac cells’ reliance of fatty acids consumption for energy metabolism when glucose becomes scarce. The experimental observation of NADH decrease induced by dietary restriction in the heart at tissue level has not been reported to our best knowledge. The Chance redox scanner demonstrated the feasibility of 3D imaging of the mitochondrial redox state in the heart and provides a useful tool to study heart metabolism and function under normal, dietary-change and pathological conditions at tissue level.

## 1. Introduction

As a complex system, the heart is heterogeneous in structure and function^1–6^ and the metabolic heterogeneities can indicate the health state of the heart. In the presence of oxygen, the cardiac muscle cells oxidize the food-derived metabolites to produce ATP in the mitochondria through the NADH/NAD^+^ couple which acts as a central electron carrier. NADH is a sensitive indicator of tissue metabolic change in response to oxygen supply.^7–9^ For example, under hypoxic and ischemic conditions, patchy heterogeneous patterns of NADH fluorescence would appear.^10–19^ One of the advantages of molecular imaging is to demonstrate the metabolic heterogeneity with a high spatial resolution and ideally at deep tissue level. However, global mapping of the metabolism of the entire heart with a high spatial resolution has not been reported.

The high metabolism of the heart requires a continuous supply of energy for the heart to function properly. The cardiomyocytes are able to use multiple substrates for their energy metabolism to generate sufficient ATP for a very high energy demand with a preference of fatty acids as up to 90% of the main energy source.^20–22^ Carbohydrate and fatty acid metabolism is tightly coupled in the heart^23,24^ and the regulation of energy metabolism is complex and involves the change in the mitochondrial redox state (NADH/NAD^+^ ratio).^22,24^ However, it is unclear how the mitochondrial redox state responds to the switch from carbohydrate and fatty acids to fatty acids only, and it has not been reported how the mitochondrial metabolic state of the heart responds to food deprivation of the host or fasting.

*In vivo* or *ex vivo* imaging study of the metabolic response of heart to fasting has significance since the beneficial effects of caloric restriction (CR) or intermittent feeding (IF, reduced meal frequency) on health has been widely studied. It has been demonstrated that CR or IF extends lifespan accompanied by a change in NAD^+^ and/or NADH level as well as NADH/NAD^+.25–27^ It was also shown that CR and IF have beneficial effects on cardiovascular system^28^ and fasting protects against myocardial ischemia.^29–33^ It is known that the change of NADH and NAD^+^ in response to food deprivation is tissue specific.^27^ However, it is unknown how they would change in the heart tissue.

In the present study, we employed the redox scanning technique^34–38^ to investigate how the mitochondrial redox state of heart tissue changes when the animals were under an overnight fasting. This technique is based on collecting the endogenous fluorescence of NADH and Fp distribution across a tissue section at the liquid nitrogen temperature using the Chance redox scanner to generate images of the mitochondrial redox indices (NADH, Fp and their ratios), followed by the quantitative assessments. The fluorescence properties of NADH and Fp are good indicators of tissue metabolism, and particularly useful for probing the metabolic processes occurring in the mitochondria. It was shown that NADH/Fp is correlated to the mitochondrial redox potential NADH/NAD^+^ (see Refs. 34 and 39) which correlates to the thermodynamic potential Δ*G* of respiratory chain in the mitochondria.^40^ NADH fluorescence photography applied to study the heart tissues under ischemic and perfused conditions showed that ischemia raises NADH level a few folds.^15–17^ Here we present the preliminary imaging results with three-dimensional (3D) sub-millimeter resolution of the spatial distribution of the mitochondrial redox state in both the normal and fasting hearts, and show the quantitative effects of host starvation on the metabolic function of mitochondria in rat hearts. We will also show the regional patterns of the mitochondrial redox state across the cardiac chambers which has not been reported.

## 2. Materials and Methods

### 2.1. Animal preparation

The animal protocol was approved by the Institutional Animal Care and Use Committee at the University of Pennsylvania. For the present study, total of seven heart samples were collected. Six female Sprague Dawley rats were randomly divided into two groups (*n* = 3 for each). The fasting group went for fasting for 14.5 h from 8:30 pm–11:00 am. The control group was normally fed. The seventh rat was also fed normally. All seven hearts of the anesthetized rats were resected and immediately placed in liquid nitrogen within a few seconds after removal. All of the organs were stored in liquid nitrogen.

### 2.2. Sample preparation for redox scanning

**Fig. 1:**
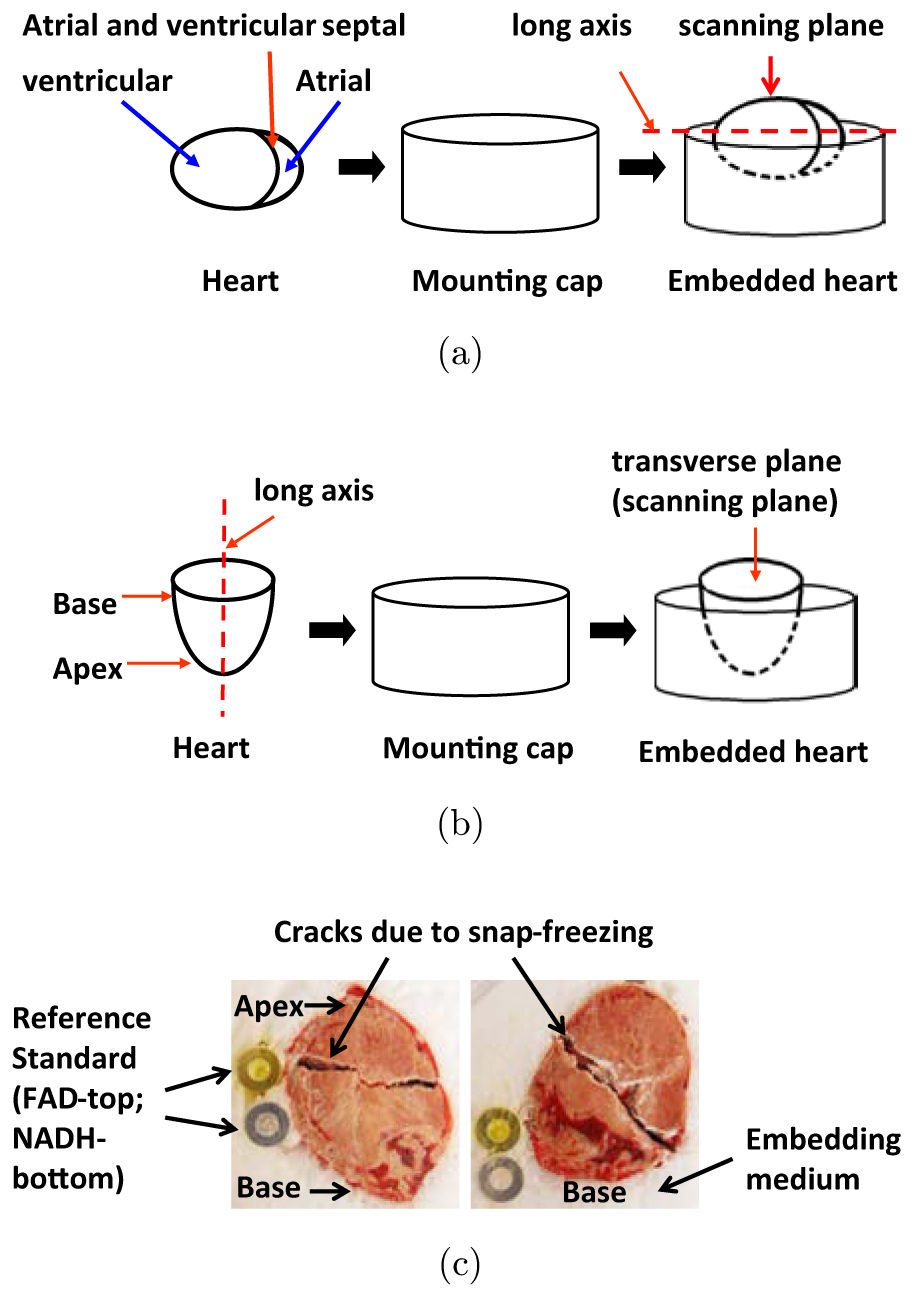
Sample embedding orientations (a) and (b) and the photos of the last scanned sections of the rat hearts embedded in the way as shown in (a), where on the left is the control heart corresponding to Fig. 2 and on the right is the fasted heart corresponding to Fig. 3.

Organ embedding for redox scanning was conducted the similar way as previously described for tissues.^41–43^ Briefly, six hearts were embedded with the long axis lying horizontally in parallel with the plane of scanning as shown in Fig. 1(a). Two reference standards (FAD and NADH in Tris-HCl buffer of pH 7 with a concentration of 500 and 100 *μ*M, respectively) were inserted next to the tissue. The seventh heart specimen was excised into two portions. The apical portion (~ 9.4 mm long from the cutting plane to the apex) was used for the present study and was snap-frozen immediately after the excision. Different from the aforementioned embedding orientation, this heart was embedded with the long axis of the heart perpendicular to the scanning plane as shown in Fig 1(b). The entire specimen was exhausted by consecutive scanning spacing of 400 *μ*m.

### 2.3. Redox scanning

The embedded samples were first shaved flat by the miller of the Chance redox scanner to just expose the top tissue. This was taken as the top section and the starting plane of the specimen. About 3–5 sections spacing 800–1000 *μ*m at various depths (400–3200 *μ*m from the top section) were scanned for each of the six hearts with an in-plane spatial resolution of 200 *μ*m. The optical filters were 365BP26 (Ex) and 455DF70 (Em) for the NADH channel and 440DF20 (Ex) and 515DF30 (Em) for the Fp channel. Neutral density filters were used in the emission channels when scanning the reference standards and were taken into account for the quantitative analysis of the imaging data.

### 2.4. Data analysis

The acquired data were further processed using a customized Matlab^®^ program to obtain the mean values of the redox indices (Fp, NADH and NADH/Fp) and their standard deviations (SDs) for each section. Pixels with SNR > 7.5 for both Fp and NADH channels were included for the analysis. The nominal concentrations of both Fp and NADH were obtained by calibrating their raw signals to the signals of the corresponding reference standards embedded adjacent to the heart tissue and expressed in *μ*M. Note that the nominal concentrations do not necessarily reflect the true concentrations of the tissue. The purpose of using the reference standards is to exclude the day-to-day instrument variations and to compare samples scanned at different time. The section mean value of a specific redox index was first obtained by averaging all pixel signals of that section. The statistical analysis was performed for all the redox indices and their SDs using the univariate analysis model in SPSS (v20) with each of the indices as the dependent variable and the feeding status as the independent variable and the section depth as a covariate (*p* < 0.05 is considered statistically significant). The univariate analysis result was further confirmed by the multivariate model.

## 3. Results

### 3.1. Redox heterogeneity in the hearts

Metabolic heterogeneity is an essential property of heart tissue. Altered metabolic heterogeneity can occur in pathological states, such as hypoxia and ischemia.^14,18,19^ It is not known if and how fasting would alter the redox heterogeneity of heart tissue. We scanned the successive sections of the hearts in the fast and control groups with an in-plane resolution of 200 *μ*m and section spacing of 800–1000 *μ*m. We have observed sub-millimeter heterogeneity in NADH, Fp and redox ratio images of both the normal and fasted hearts.

**Fig. 2:**
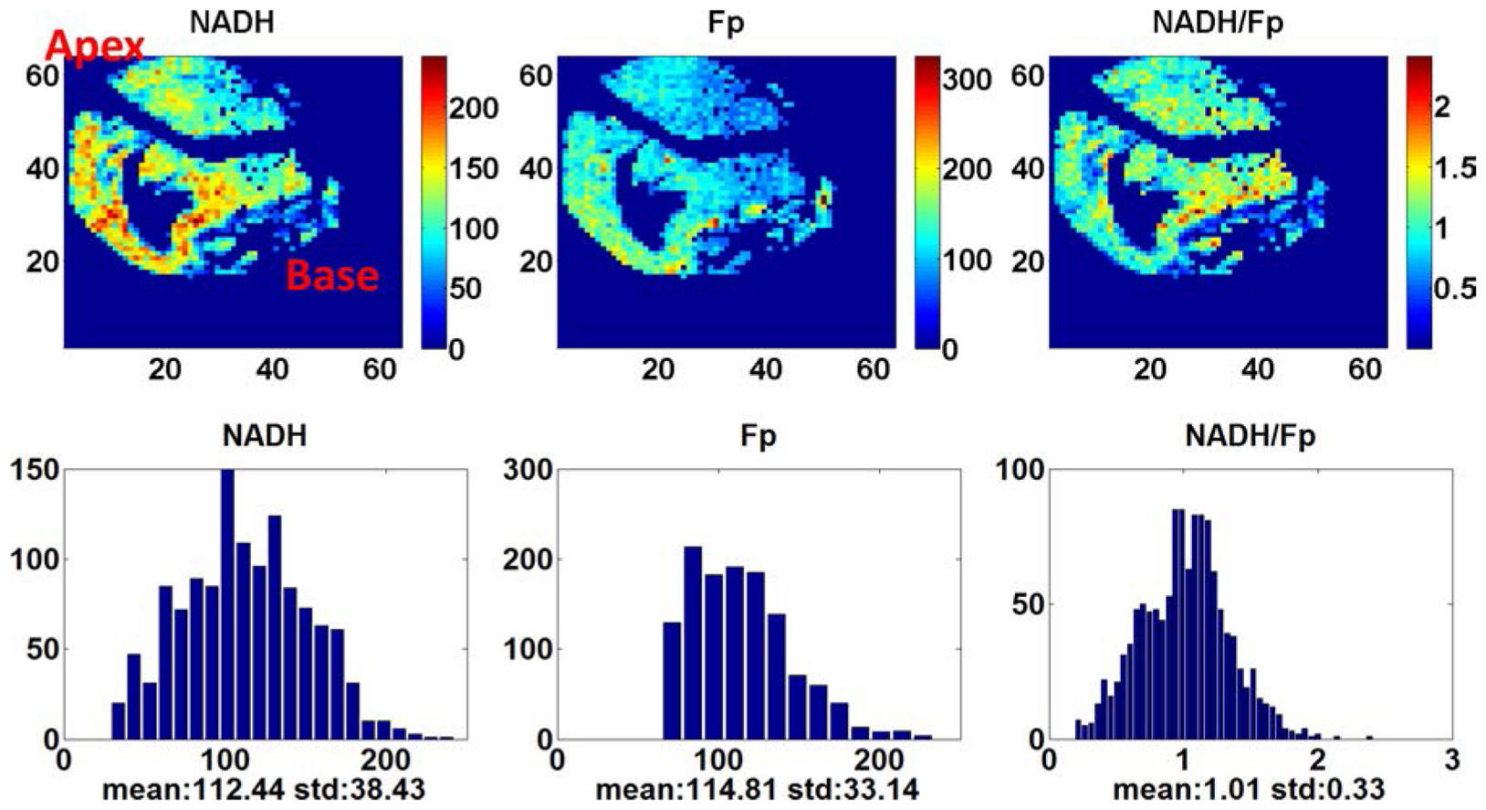
The typical heart redox images of the normally fed rats embedded in the way as shown in Fig. 1(a), section depth: 1600 *μ*m.

Figure 2 shows the typical pseudo-colored redox images of a heart section of a normally fed rat. As reflected in the color variations and the histogram display of the distribution of the redox indices, the mitochondrial redox state is heterogeneously distributed across the section. For this particular section (depth: 1600 *μ*m), the area near the base appeared to be more reduced and the majority of the cells had redox ratio NADH/Fp centered around 1 (seen from the corresponding histogram). Micro-heterogeneity of sub-millimeter size is also clearly seen, particularly near the base area. The area that has no NADH or Fp fluorescence signals is the atrium and ventricle.

**Fig. 3:**
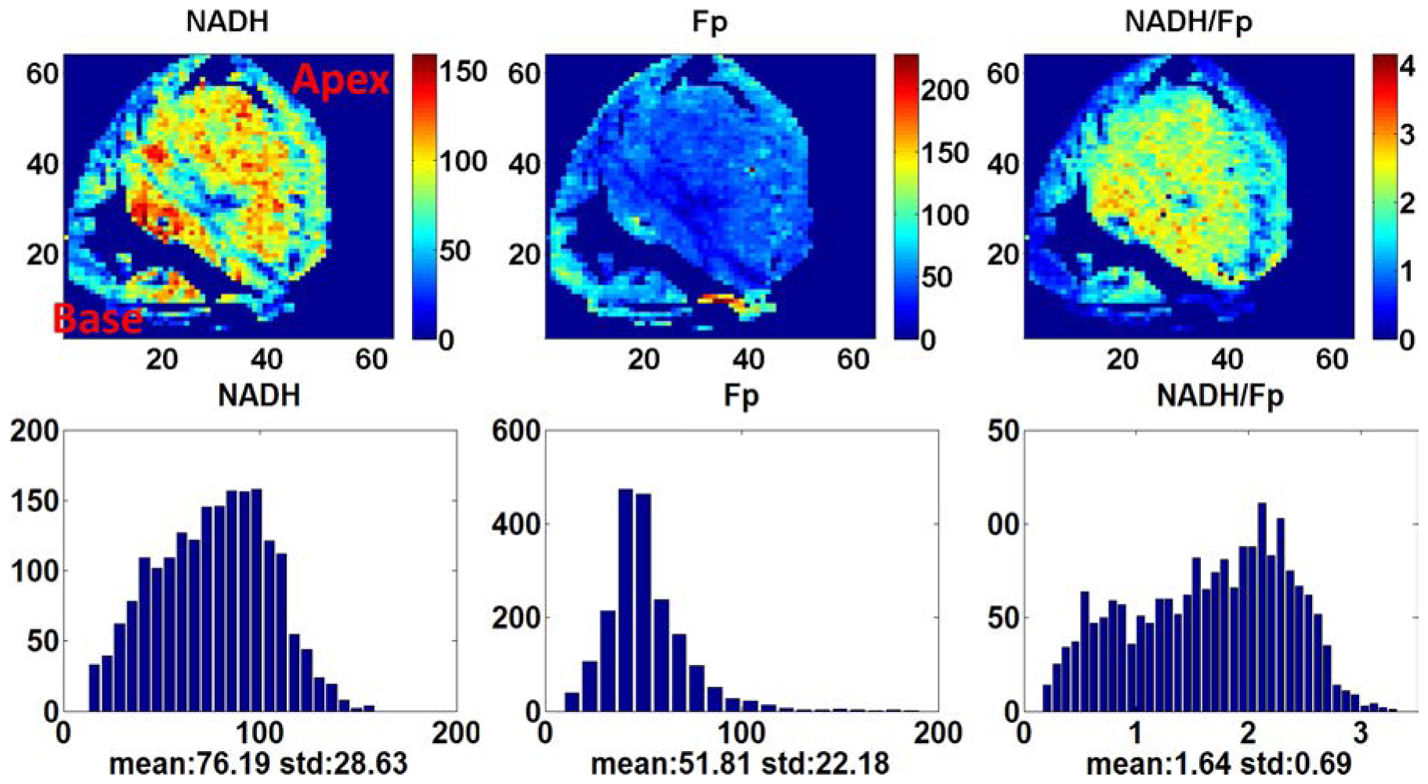
The typical heart redox images of the fasting rats embedded in the way as shown in Fig. 1(a), section depth: 2300 *μ*m.

Figure 3 shows the typical pseudo-colored redox images of a heart section of a fasting rat. For this section (depth: 2300 *μ*m), the central area appeared to be more reduced and the cells roughly appeared to be in two different redox states with average redox ratios centered around 0.8 and 2, respectively (seen from the corresponding histogram). Micro-heterogeneity of sub-millimeter size is also seen. The area without NADH or Fp fluorescence signals near the base is the atrium and the one near the apex is the ventricle.

**Fig. 4:**
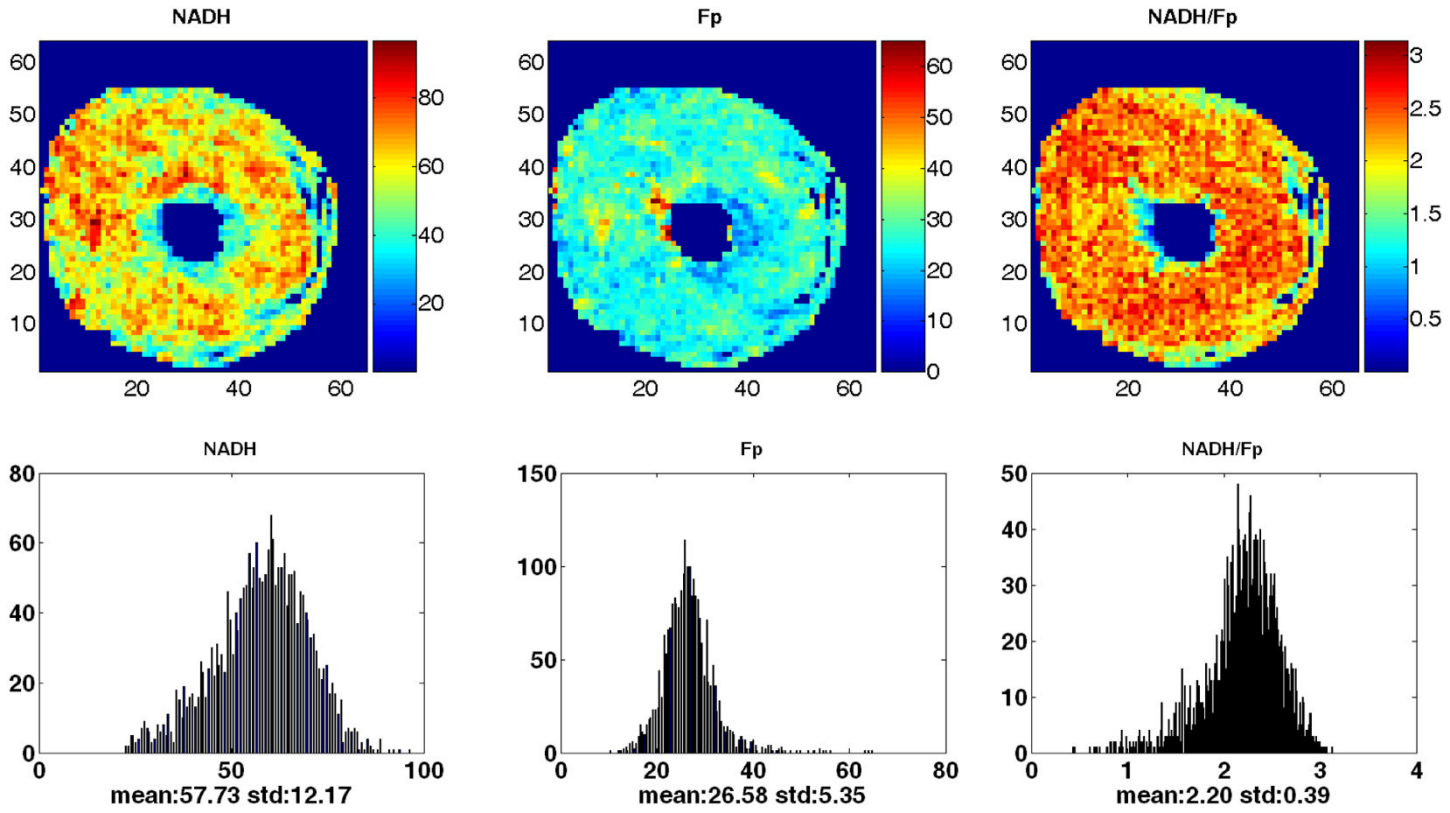
The redox images of the additional normal heart at 2200 *μ*m [a transverse plane, embedded in the way as shown in Fig. 1(b)]. Spatial resolution: 200 *μ*m.

**Fig. 5:**
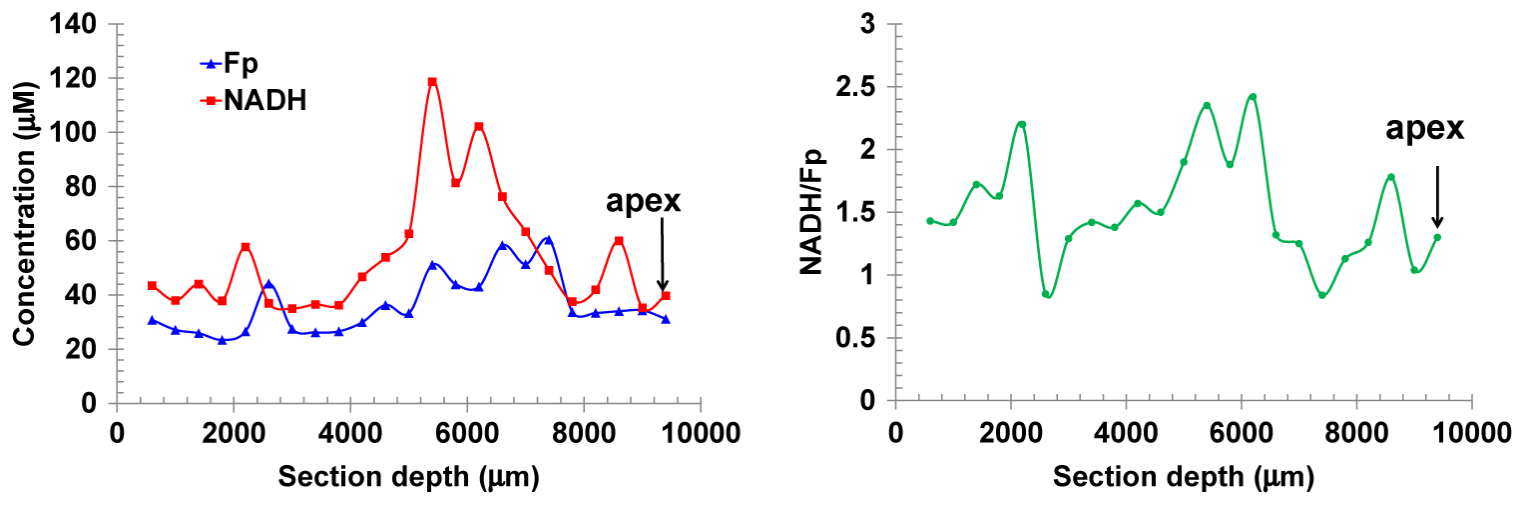
The variation of the redox indices along the long axis of the normal heart embedded in the way as shown in Fig. 1(b).

As we have not seen any report on the 3D mapping of the heart redox state, we exhausted the entire heart by successive scanning of the transverse plane of the seventh heart (normally fed, embedded with the long axis perpendicular to the scanning plane). Figure 4 is the section at depth of 2200 *μ*m from the starting plane near the base. The mean values for each section were plotted against the section depth as shown in Fig. 5. Comparatively speaking, NADH varies more than Fp with the largest variation of ~ 90 *μ*M at depth of ~ 5200 *μ*m. From the distribution of the redox ratio NADH/Fp, sections at depth of 2000, 5200, 6200 and 8600 *μ*m were more reduced, whereas sections at depth of 2800 and 7200 *μ*m were more oxidized.

### 3.2. Reduced redox heterogeneity level in the fasted hearts

Using the SD as a measure of the tissue heterogeneity level, we found that the SD of both NADH and Fp significantly decreased after 14.5 h fasting (see Table 1), indicating that fasting made the heart metabolically more homogeneous.

### 3.3. Reduced NADH content in the fasted hearts

The response of NADH to dietary change is tissue specific^27^ but it has not been reported how it changes in the fasted heart. As shown in Table 1, in the fasting group, the hearts had significantly less NADH compared to the control group (*p* = 0.038), indicating that 14.5 h fasting caused the rat hearts to contain 28.6% less NADH. The Fp level showed no statistically significant change after fasting. Additionally, NADH/Fp decreased due to fasting with a borderline level of statistical significance (*p* = 0.076).

## 4. Discussion

In this study, we measured the endogenous fluorescence signals of NADH and Fp from snap-frozen rat hearts for investigating how NADH, Fp and the cellular redox state (NADH/Fp) of the heart responded to host food deprivation. As the cytoplasmic NADH contribution is negligible compared to the mitochondrial NADH signal in the heart cells^19,44,45^ and NADH concentration is 2–4 times that of NAD(P) H,^46,47^ the blue fluorescence signal we collected is interpreted as mainly mitochondrial NADH.^19,48^ It was suggested that the NADH bound to Complex I contributes significantly to the NADH fluorescence.^49^ The Fp signal also originates mainly from mitochondria.^37^ Although a nonmetabolic component cannot be excluded in the Fp signals, the metabolic sensitive Fp signals are mainly contributed by FAD-containing flavoproteins including pyruvate dehydrogenase, *α*-ketoglutarate dehydrogenase and electron transport flavoproteins (ETF).^50,51^ The redox ratio is a sensitive index of mitochondrial metabolism and the redox state.^52^ Fp/(Fp+NADH), NADH/(Fp+NADH), Fp/NADH or NADH/Fp have been used in previous reports. The normalized redox ratio Fp/(Fp + NADH) is less sensitive to perturbations than Fp/NADH does because the denominator is buffered by the value of Fp. We used NADH/Fp to represent the mitochondrial redox state for this study.

Local energy demand of heart tissue varies spatially and the molecular basis of this spatial heterogeneity has been under investigation. For example, the atria in rat heart contain higher concentration of glycogen than the ventricles^1^ and glucose transporter expression has differential regional heterogeneity.^53^ The regional pattern of glucose transporter protein expression was chamber-specific and different between the healthy and the diseased heart.^53^ The redox state is another key regulator of the mitochondrial bioenergetics in the heart. The redox micro-heterogeneity has been intensively investigated by Chance and other groups using the perfused heart models by measuring tissue NADH fluorescence under various oxygen supply conditions.^11–16^ Hypoxia or ischemia of the heart tissue causes heterogeneous zones of high NADH surrounded by apparently normal aerobic tissue. However, we have not seen published data on mitochondrial redox spatial distribution in 3D in the heart. In this study, we observed local heterogeneities of NADH, Fp and NADH/Fp ratio in both the longitudinal and transversal section of the heart. By consecutively scanning the normally fed heart from the base to the apex, we also discovered a large redox variation (see Fig. 5). Further work is needed to investigate whether and how the local heterogeneous distribution of the redox state may be related to the cardiac bioenergetics and functions. It would also be interesting to study further if and how glucose transporter expression is related to the redox state distribution. The detection of the local metabolic heterogeneity with sensitive molecular imaging biomarkers/techniques such as the redox scanning is valuable for studying heart physiology and pathology.

This study demonstrated various metabolic changes in the heart induced by dietary restriction. Our imaging data show that fasting reduced the level of redox heterogeneity in the heart as indicated by the significant decrease in the SD of both NADH and Fp mean values. The SD of NADH/Fp also has a decrease trend. The exact meaning of the more homogeneous redox indices is not clear. In future, we can perform texture analysis to find out if any particular new texture patterns arise for these redox indices in the fasted heart tissue.

We also observed that the overnight fasting caused the heart to contain less NADH. Because the ATP content in the heart is very low, to meet the very high energy demand of the heart, ATP must be generated at a high rate continuously to ensure the contractile activity of the heart. Over 95% of ATP in the normal heart is produced from oxidative phosphorylation in the mitochondria by using a variety of carbon substrates as sources. However, in well-oxygenated hearts, fatty acids are preferred energy source which provides 60–90% of the total energy where *β*-oxidation of long-chain fatty acids contribute up to 70% of energy.^21,22^ The *β*-oxidation cycles and the subsequent TCA cycles yield both NADH and FADH_2_ which then enter the electron transport chain to generate ATP. Under fasting, the circulating fatty acid levels are elevated.^21,23^ By burning fat as compared to carbohydrate, less intermediate NADH per ATP is produced.^54^ Our observation of less NADH content in the fasting group is consistent with increased reliance on fatty acid oxidation during fasting.

We observed no significant change in Fp that resulted from the overnight fasting. Fp signals originate from multiple sources. Although a shift of metabolic flux to fatty acid *β*-oxidation may increase the Fp signal from TCA cycles (less electron flux and FAD reduction), it may decrease the Fp signal from ETF as more electrons flow through from the *β*-oxidation. Thus, the total Fp signals could remain little changed as we have observed.

The regulation of fatty acid metabolism of heart tissue is complex and different from other tissues in many aspects.^22,23,55^ Studies showed that the *β*-oxidation rate is driven by demand and diminishing NADH level increases *β*-oxidation.^56–58^ Our observation of decreasing trend of NADH/Fp in the fasting group seems to be consistent with more fatty acids oxidation during fasting, but is likely to be the downstream effect, rather than the upstream cause.

Diet-induced metabolic changes that affect NADH and NAD^+^ levels have been intensively studied in order to understand the observed benefits of lifespan extension from CR. For example, it was shown that reducing glucose decreases NADH level while NAD^+^ level remains unchanged in yeast.^26^ However, for CR mammals such as mice, NADH level can either elevate or drop depending on tissue type. NADH in the skeletal muscle responds to CR with a slight decrease but it increases in both the liver and white fat tissue.^27^ The impact of CR or IF on NADH level in the fasted heart has not been reported. We observed a decrease in NADH level in fasted hearts. Mapping the 3D distribution of NADH, Fp and the redox state in the heart under fasting conditions may provide useful information that can help better understand the effects of CR on physiology.

There are a few limitations of the preliminary study. First, the sample size is small. Although the univariate analysis method with tissue depth as a covariate improved our ability to achieve statistical significance (better than the *t*-test), the high inter-and intra-sample variability make it difficult to obtain statistical significance for the redox ratio. We will include more hearts in future to test if the NADH/Fp redox ratio changes significantly as a result of fasting. Secondly, the nominal concentrations of NADH and Fp may not reflect the true tissue concentrations and the redox ratios. The fully quantitative calibration of the tissue concentrations and the redox state has been a challenge and should be addressed in future. Another one is the difficulty to clearly identify the structure of the heart due to tissue deformations and cracks caused by the direct snap-freezing in liquid nitrogen. We shall use chilled isopentane prior to liquid nitrogen to achieve a more uniform freezing of the tissue which may allow a better identification of the structure of the heart. The lowest in-plane spatial resolution of the Chance redox scanner is 50 *μ*m. For higher spatial resolution imaging and fasting imaging, we may need to use a CCD-based redox imager^59,60^ to construct 3D maps of the heart redox state at the resolution of a few microns. However, point excitation such as used by the Chance redox scanner avoids the potential fluorescence crosstalk between the pixels, which may occur when the excitation light beam has a large cross section.

## 5. Conclusions

In this paper, we reported the preliminary imaging data on the 3D mitochondrial redox state distribution of the rat heart under normal feed or fasting conditions with sub-millimeter spatial resolution. We also reported the quantitative imaging data on how fasting affects the heart redox state. Our preliminary results showed that the fasted hearts became relatively more homogeneous in the redox state and contained less mitochondrial NADH. These results provide 3D cardiac mitochondrial metabolism information with sub-milliliter spatial resolution at tissue level for the first time, which provides more insights into the mitochondrial metabolism of the rat hearts under normal and fasting conditions, and may help understand the physiological effects of CR or intermittent fasting on the heart.

## Figures and Tables

**Table 1:** The mean values of the redox indices (mean ± SD) and that of their standard deviations (mean ± SD) as well as the statistical analysis using SPSS’s GLM (univariate model) with each of the indices as the dependent variable, “fast” as the independent variable and tissue depth as a covariate. *N* stands for the number of heart samples and *S* stands for the number of scanned sections.

	NADH (*μ*M)	Fp (*μ*M)	NADH/Fp	SD_NADH (*μ*M)	SD_Fp (*μ*M)	SD_NADH/Fp
Ctrl (*N* = 3, *S* = 11)	70 ± 28	55 ± 36	1.66 ± 0.75	25 ± 14	22 ± 17	0.55 ± 0.31
Fast (*N* = 3, *S* = 13)	50 ± 14	45 ± 15	1.21 ± 0.36	16 ± 5	12 ± 5	0.41 ± 0.16
*p*	0.038	0.42	0.076	0.045	0.032	0.16
